# Using the Socio-Ecological Model to Explore Parents’ Resilience and Perceptions of Adverse Childhood Experiences: A Qualitative Study in the Southeastern United States

**DOI:** 10.3390/healthcare14101414

**Published:** 2026-05-21

**Authors:** Maribel G. Dominguez, Christine Markham, Andrew E. Springer, Louis D. Brown

**Affiliations:** Department of Health Promotion and Behavioral Sciences, The University of Texas Health Science Center at Houston School of Public Health, Houston, TX 77030, USA; christine.markham@uth.tmc.edu (C.M.); andrew.e.springer@uth.tmc.edu (A.E.S.); louis.d.brown@uth.tmc.edu (L.D.B.)

**Keywords:** adverse childhood experiences (ACEs), resilience, generational trauma, socio-ecological model (SEM)

## Abstract

**Highlights:**

**What are the main findings?**

- Explores parental resilience as a protective factor against adverse childhood experiences (ACEs).
- Parent support groups as an inter-socio-ecological model level to improve a parent’s resilience through lowering isolation, increasing perceived social support, and improving community connectedness.

**What are the implications of the main findings?**

- Strength-based, trauma-informed engagement models are essential to informing multi-level strategies for ACE prevention and mitigation.
- Multi-level interventions should target resilience within relational systems by examining parents’ perspectives through a social–ecological model (SEM) framework within a trusted community-based organization serving children’s and families’ mental health needs.

**Abstract:**

**Background:** The negative impact of adverse childhood experiences (ACEs) on child development is documented. The parent–child relationship protects against ACEs and improves healthy child development, playing a crucial role in preventing and mitigating ACEs by strengthening parental resilience. However, there is a gap in the literature on our understanding of parental resilience’s impact on the parent–child relationship within the social–ecological model (SEM) (i.e., intra- and interpersonal, community, and societal levels). **Objective:** This study explores parents’ perspectives on parental resilience as a protective factor for preventing and mitigating ACEs at every level of the SEM. **Method:** This study uses a thematic analysis approach for qualitative research. In-depth individual interviews (n = 21) were conducted with members of a parent support group (PSG) (85% female) based in a community-based organization serving families. Demographic information and ACE scores were collected for each participant to describe the sample. **Results:** Key findings highlighted parents’ perspectives on improved resilience through self-regulation and social support following participation in PSGs, conceptualized as an inter-level construct within the SEM mechanism due to its influence on parents’ well-being, traversing SEM levels. Under Theme 1: The Many Faces of Parental Resilience, Theme 3: The Power of Close Relationships, Theme 4: Community Resources as a Buffer, and Theme 7: Change Through a Policy Lens: “Anything that protects them,” parents expressed a strong desire for ACE prevention and mitigation strategies and called for systemic policy change to combat ACEs. **Conclusions:** Parental resilience perceptions are valuable and hold promise to inform the future institutionalization of a multi-level parent resilience-focused framework, which will aid in ACE prevention and mitigation.

## 1. Introduction

Adverse childhood experiences (ACEs) are potentially traumatic experiences in a child’s life or family environment [1,2,3,4]. Traditionally, there are 10 ACEs included in three major categories: neglect, abuse, and household dysfunction [3,4]. Children of parents with four or more ACEs are 3.25 times more likely to also experience four or more ACEs compared to children of parents without ACEs [5]. Parents with greater childhood adversity are at risk of reduced parenting capacity, with a greater likelihood of showing behaviors of hostility, withdrawal, stress, and reduced responsibility and sensitivity [6]. Thus, ACEs may be linked to cross-generational parenting practices from parents to children (i.e., cross-generational transmission of adversity) [5,6,7]. Thus, parental resilience serves as a protective factor (PF) in the parent–child dyad, decreasing the odds of ACE transmission in children [5].

### 1.1. Exploring Resilience as a Protective Factor Against ACEs

Resilience is a complex construct described as a PF that helps individuals overcome adverse or traumatic events [8,9]. PFs can be external or internal protections against poor mental health [10,11]. External PFs are linked to social bonds, including the family supportive network, community (e.g., community involvement), school (e.g., school connectedness), and peer interactions (e.g., prosocial peer relationships) [12]. Internal PFs relate to personal factors such as social skills, sense of future orientation, emotional insight, positive self-concept, and empathy [11]. Parents represent a child’s first significant social relationship [13,14]. Thus, examining parents’ perspectives of factors that promote resilience may benefit parents and children in mitigating the adverse outcomes of ACEs.

### 1.2. Socio-Ecological Model of the Parent–Child Relationship

The socio-ecological model (SEM) offers a framework for identifying factors that shape parent-to-child resilience in response to ACEs across various ecological levels of influence, ranging from the individual to the policy level [9,15]. The CDC’s four-level SEM for violence prevention provides a structure for integrating ACEs by situating ACE history at the individual level while also recognizing its interactions with interpersonal, community, and societal risk and protective factors [16]. The intrapersonal level (i.e., individual) includes knowledge, attitudes, behavior, and skills (i.e., emotional control) for both parents and children [17,18]. Emotional expression and communication are linked to enhanced social competence and mental health [18]. Factors related to personal history, biology, attitudes, beliefs, or behaviors are also at the individual (intrapersonal) level [18]. The interpersonal level focuses on social networks, including families and friendships [17,18]. A child–parent network environment influences intrapersonal responses (i.e., social behavior) [9,19]. For example, family conflict (i.e., household problems) is linked to low child prosocial behavior and child adjustment problems [19]. Close relationships also play a role in both increasing vulnerability or risk and/or serving as a PF against violence exposure [20]; relationships can include friends, peers, or family members [18]. Parenting factors (for example, parent–child communication) or family mentoring (promoting positive peer norms and healthy relationships) may help improve the quality of close relationships [18].

The community level (which contextualizes early social relationships) focuses on organizations and social institutions [18]. Schools, workplaces, and neighborhoods encompass the community level and provide important context for observing and assessing the prevalence of environmental violence and trauma [18]. Improving environmental settings (e.g., safe places to live, learn, work, and play) and addressing neighborhood poverty and instability could help prevent violence [18].

The public policy level (i.e., societal) focuses on the local, state, and national regulations that influence health, education, economy, and social policies [18,21,22]. Globalization-related regulations, such as economic migration, influence a child’s perception of parental authority (i.e., the acceptability of disagreeing with parents) [9]. The factors related to the policy-level impact of the socio-ecological model include norms (society or culture) that contribute to inequality, health, education, economic conditions, and social policy [18].

### 1.3. Significance of Qualitative Research in the Study of ACEs

Studies have established the importance of the parent–child relationship for healthy child development, but research is limited on how factors at different levels of the socio-ecological model impact the parent–child relationship. The socio-ecological model provides a useful framework for examining parental resilience influences across multiple environmental levels [23]. Exploring parents’ perspectives on how to promote resilience may help develop a socio-ecological framework of parental resilience that explains barriers and solutions within the societal network. Focusing on the multiple levels of influence can broaden the options for interventions and environmental changes [24]. This study aims to explore parental perspectives on how to promote resilience among parent–child dyads who seek to do so across levels of the socio-ecological framework, especially in response to ACEs. The present study addresses a qualitative research gap by utilizing the SEM framework to capture parent perspectives and thus add a building block to a future SEM parental resilience framework for preventing and mitigating ACEs. The findings aim to inform the development of a parent-resilience framework to mitigate ACEs and promote parental ER.

Research Question: Among parent-support-group-seeking parents, what helps and hinders their parental resilience, and how is their resilience shaped by factors at each level of the socio-ecological model?

## 2. Materials and Methods

We utilized the Consolidated Criteria for Reporting Qualitative Research (COREQ), a 32-item checklist for interviews and focus groups, to organize the investigation’s content and write up the findings. This study was reviewed and approved by the Institutional Review Board (IRB) of The University of Texas Health Sciences Center at Houston (UTHealth Houston). All parents were given an informed consent form that outlined the study’s aims and emphasized the voluntary and confidential nature of the study.

### 2.1. Interviewer and Coder Personal Characteristics

The lead researcher scheduled and conducted all interviews from information provided by a community-based mental health organization serving children and families in the Southeastern part of the U.S. The research team’s experience with public health research focused on adolescent and adult mental health. All authors hold doctoral degrees in psychology or public health, with extensive experience in qualitative research methods.

### 2.2. Relationship with Participants, Knowledge, and Characteristics

The participants and researchers had no prior relationship. Interviewer characteristics were disclosed to the participants, including the possibility of bias, assumptions, and the researchers’ interest in the research topic.

### 2.3. Theoretical Framework

The study used the CDC social–ecological model to explore and organize parent-reported factors that influence resilience by ecological level (individual, interpersonal, organizational, community, and societal [16]) to examine the role of building parenting resilience concerning ACEs. The methodological orientation underlying this study is inductive coding, which facilitates the identification of patterns, categories, and themes (Creswell & Creswell, 2016) [25].

### 2.4. Participant Selection

Sampling: Participants were selected via purposive sampling (i.e., purposefully recruited from this group). They shared the characteristic of having participated in the parent support group program, indicating the caregivers had a general interest in building caregiver practices, healthy relationships with their children (i.e., daily routines), resiliency, buffering toxic stress with age-appropriate best practice strategies, and obtaining ongoing support in their parenting.

Study Recruitment: Participants were approached via an email from the community-based organization’s staff, detailing study eligibility, the center’s support, and the study’s purpose, along with a flyer and sign-up form that included this information. Participants who expressed interest via the sign-up form received a phone call from the lead researcher within 3 days. If the call went unanswered, the lead researcher sent a follow-up text immediately. Outreach efforts were repeated after a week without response with a second reminder email from the community-based organization.

Sample size: The total interview sample included n = 21 (interview-level breakdown: 18 mothers and 3 fathers) participants from the parent support group program (survey-level breakdown: 17 mothers and 3 fathers). Data saturation was reached at 21 interviews, indicating robust, value-added insights. One parent responded but did not complete the demographic survey.

### 2.5. Setting

The data were collected via online video communication platforms, including Zoom, Microsoft Teams, and FaceTime on iPhones, depending on the participant’s preference. Interviews were video recorded for verbatim transcription.

### 2.6. Study Measures and Data Collection

Post-Interview Demographic Survey: Participants completed an electronic post-interview survey that included ACEs and demographics, including age (in years), gender, race/ethnicity, education level, and caregiver status.

Adverse Childhood Experience (ACE) Questionnaire: A 10-item, closed-ended (i.e., yes/no) questionnaire developed by Felitti et al. (1998) [4]. The questionnaire covers household dysfunction (e.g., mental illness, domestic violence, parental substance abuse, parental separation or divorce, incarcerated household members), abuse (physical abuse, emotional abuse, sexual abuse), and neglect (physical neglect, emotional neglect). Participants were considered to have high ACEs if they responded “yes” to four or more questions, and those with three or fewer “yes” responses were classified in the low-ACE category.

### 2.7. Interview Guide

The parent-resilience interview guide was developed by the lead researcher, using the SEM as a framework, with the aim of exploring parent input on parent resilience at each SEM level. The overarching aim of the guide was to explore the factors that influence parent resilience. The interview guide included the researcher’s introduction, important disclosures to participants about voluntary participation, and interview questions. We organized the interview guide questions using the socio-ecological model, as informed by the work of Krug et al. (2006) [26] and the ACE domains identified by Felitti et al. (1998) [4]. Three pilot interviews were conducted with community members who identified as caregivers.

Interviews: No repeat interviews were conducted. Each interview was video-recorded. If a participant expressed discomfort with being video-recorded, we opted for audio recording only (one participant preferred audio-only recording). Interviews lasted 40 to 60 min. One parent chose to skip the interview section on policy and culture, and another parent opted not to answer the question regarding individual resilience.

Compensation and records: Free mental health counseling resources were shared with each participant at the end of the interview. The participants received a USD 20 Walmart electronic gift card as compensation. Field notes were used to track special circumstances, such as the occurrence of Hurricane Milton, which caused parental distress, rescheduling, and cancellations. Participants were not provided transcripts or feedback on the findings. Participant IDs were generated randomly, and the original numbers were de-identified.

### 2.8. Data Analysis

Our goal was to examine parental perspectives on parental resilience as a PF for preventing adverse childhood experiences and supporting positive childhood experiences, using the socio-ecological model as a framework. We used a demographic survey with ACE measures to describe the parent sample.

This qualitative study was guided by a thematic analysis, using deductive coding to organize and interpret the data in line with the SEM. The codebook was developed by one coder, and additional or new codes were added as interviews were coded. Thus, themes and subthemes were organized according to the levels of the socio-ecological model through an iterative, deductive, and inductive coding process.

In the first step, we deductively established the SEM framework (i.e., step 1: SEM framework) by developing interview guide questions effectively aligned with the SEM level. For example, for the interpersonal-level questions, we let participants know that “This level includes close relationships (peers, partners, or family members), along with parenting (parent–child communication) and family mentoring (promoting positive peer norms, promoting healthy relationships),” followed by asking, “What strategies or activities do you think would help a parent or caregiver improve their relationship with their child?” (The interview guide can be found in Appendix A). Following this, in step two, we adopted an inductive approach, during which all codes were identified from the actual interview data (i.e., step 2: Identifying all codes from collected data) (Hayre & Zheng, 2021) [27]. We specifically employed the inductive approach, as described by Creswell and Creswell (2016) [25], working back and forth between themes until we established a comprehensive set of themes derived from codes that helped us explore perspectives on factors associated with parental resilience.

Finally, in step three, we organized the codes and emerging themes by utilizing a four-coding system (step 3: SEM value coding organization): the first coding level was “value coding” at the individual level (i.e., a technique to categorize data relating to attitudes, beliefs, or personal values), implemented to identify participant culture or link to resilience at the individual level. The second value coding was a “hybrid value coding,” with deductive codes established a priori (i.e., in advance for each level as responses that fit directly to the questions organized by SEM in the interview guide) and followed by an inductive process (i.e., at each level of the socio-ecological model, we assessed keywords to extract relevant codes). The first hybrid coding level was the interpersonal deductive codes, which included “friends” and “family.” The second hybrid coding level was community value coding, with deductive codes including “schools” and “neighborhoods.” Finally, the third hybrid coding policy level included the deductive codes “society,” “law,” and “policy.” This structured, framework-based qualitative analysis was intended to ensure the participants’ perspectives were effectively organized within the environmental system.

Software: We utilized the qualitative data analysis software by ATLAS Scientific Software Development GmbH (version 26.0.1) and Microsoft Excel. Interviews were transcribed using automated tools when available; otherwise, manual transcription was employed.

## 3. Results

### 3.1. Sample Characteristics

The interview sample consisted of 18 mothers and 3 fathers (n = 21 completed the interview portion), aged 25–48 years, who voluntarily joined a parent support group (descriptive characteristics are shown in Table 1). One-half of the sample was Hispanic (n = 10; 50%), with 80% White (n = 16) and 30% African American (n = 6) participants. Participants were allowed to select multiple ethnic and race categories, including Hispanic/Latino, in addition to a racial category. The majority of parents had an ACE score of less than 4 (n = 15; 75%), with three parents reporting zero ACEs.

### 3.2. Qualitative Results

Parents identified areas of resilience and emotional support, along with recommendations. The following section presents the qualitative results, organized by SEM level into themes and subthemes. Seven major themes were identified (the definitions of each theme and subtheme are presented in Table 2). The parent support group (PSG) was conceptualized as an inter-level construct within the SEM, as participants described its positive influence from the individual (e.g., coping and self-efficacy), interpersonal (e.g., peer support), and community (e.g., social connectedness) levels. Representing PSG across these levels reflects the SEM’s emphasis on cross-level interactions and highlights how a single intervention, such as PSG, can simultaneously shape multiple layers of parental resilience.

#### 3.2.1. SEM Individual Level

- Theme 1: The Many Faces of Parental Resilience

This overarching theme captures the parents’ internal fortress of inner strength and, thus, emotional resilience shaped by their evolving experiences. The patterns in this theme suggest that parents draw upon various faces to navigate emotional challenges and adapt to an evolving parenting journey. Thus, this theme explores the various activities and factors that help parents develop resilience, suggesting that inner reflection and regulation contribute to a parent’s mental well-being. The following subthemes reflect on self-managing strategies that help a parent cope and thus improve their children’s well-being.

1. Subtheme 1: Faith

The element of faith was a driving force for resilience in parents. Faith, in a higher power manifested in families with multiple parents, was spoken of confidently and clearly when describing how their faith builds their resilience. Participant 24 describes their faith as a builder of resilience: “I would say the one thing that’s definitely helped me build resilience is definitely my faith, my faith in God, number one.” Similarly, participant 13 mentioned strength through faith, stating, “I just remain strong and stay and pray and continue doing what I’m doing.” Thus, a key aspect of this subtheme is that faith went hand in hand with reason, with parents’ ability to accept adversity, and with the understanding that a higher power was in control.

2. Subtheme 2: Reflecting on their own childhood

Reflecting on one’s own childhood was an influential factor in parental resilience, as it is a mental exercise that connects the individual to the roots of their own development. Parents emphasized how they built resilience through reflection on their upbringing. Parents reflected on their own childhood experiences, processing their feelings and learning to manage them, and then narrated their childhoods all over again. Participant 11 emphasized how they focused on not repeating patterns, ones that their own parents had followed, saying, “[Original Spanish] To cope with certain situations… and in the same way, to be able to teach my daughters how to do it—not to repeat, perhaps, what my parents did or didn’t do with me.” Self-reflection is a key factor in shaping their parenting; this subtheme suggests that actively reflecting on what lifted them up would help lift their children’s life outcomes.

3. Subtheme 3: For their children

Children, or offspring, themselves play a pivotal role in their parents’ ability to cope with everyday life adversities and thus are a force in parental resilience. Parents focused on building resilience in their children and thus noted that their children are a force that helps them remain resilient. For example, participant 6 stated that resilience arises from wanting a better life for their children: “I think it comes from my kids. Like, I just really love my kids and just being able to make them happy and provide a good life for them.” Similarly, participant 15 describes their resilience and wanting to improve for their children: [Original Spanish]: “Well… love—that is, the love for my son—the desire to always give him the best has been the main motivation for me to keep looking for ways to improve myself, first of all.” This key subtheme reflects how the parents’ selfless love is shown in their willingness to provide for the good of their children—that is, by focusing on what benefits the children rather than having the children serve the parents’ own needs.

4. Subtheme 4: Emotional regulation (ER)

ER refers to the ability to manage and balance emotions to respond appropriately, and thus, it is a component of parental resilience. This subtheme captures how parents bolstered their children’s resilience by regulating their own emotions, a practice they realized was fundamental. Participant 15 describes emotion regulation as “fundamental” and “vital” to helping them navigate changes:

[Original Spanish]: “Well, for me, it has been fundamental to manage my emotions, and obviously, it’s super important to give yourself permission to feel what you feel. I think, as a parent, it’s really vital to create space for those emotions to be expressed…” Thus, this subtheme emerged around the positive impact of parents learning how to regulate their emotions, which builds space that allows for conscientious parenting, which builds resilience while also protecting the parent–child relationship.

5. Subtheme 5: PSG individual (interlevel subtheme)

Parent support groups (PSGs) offer a valuable network for parents to share experiences, resources, and advice, while also focusing on ER. As previously noted, PSGs were classified as an interlevel subtheme, with impacts at each level preserved in line with parents’ perspectives (for this reason, we have created a unique space to describe parents’ resilience in relation to PSGs in three levels of the SEM). Thus, PSGs were demonstrated to be an “interlevel subtheme,” or a factor—in this case, a support group for parents—that cannot be classified into a single level because the reported impact goes across all levels of the SEM. To present the reported positive impact of PSGs, we have categorized the PSG subthemes into individual, interpersonal, and community levels.

At the individual level, PSGs serve as an external PF factor, helping parents regulate their emotions individually. The PSG not only helped parents manage their emotions, but also helped them ground their emotions by reducing isolation and learning how to manage stress. Parent 24 described that, “…Being a part in that parent support group… you know, speaking with people that are going through similar situations and being able to express how I’m feeling as a parent and have that support from the collective, it definitely helps me cope…”

PSGs are essential to helping parents build resilience, as parents are often the closest support a child has, and ER is crucial in preventing and mitigating ACEs. While having an emotional support system has been described by parents as key in helping maintain or strengthen their resilience, some parents have a different environment without any external family or friendship support. To illustrate, participant 23 described how helpful PSGs are, especially for a parent who has no family or friend support:

“So I don’t have much support at all. I moved away from my family when I was younger, and then once I became a parent, that made it more difficult for me because I really had no support as far as family…. So that’s the part that makes things difficult for me, which is not having the support. The resources that I use to fill that void kind of would be like, this is kind of embarrassing to say, but I do join a lot of, like, social media parenting groups, and I’m able to connect with other parents who are in a similar situation, or that are just kind of more understanding of what I’m going through. So just hearing that other people are going through it helps me kind of, okay, cope with certain situations and be like, okay. This is something that I can get past. So, I use that. I try to lean on school resources. If there’s anything available, I reach out to them often just to see if there’s any way to help.”

- Theme 2: Resilience forged in scarcity

Theme 2 highlights how external structural challenges—particularly financial hardship—hinder a parent’s resilience through increased stress or burden. A key barrier to resilience at the individual level was the lack of economic resources, which was related to both “single-parent home” and the absence of “means” (i.e., economic means). Further, financial stress acts as a cognitive load, keeping parents in survival mode, weakening their coping mechanisms, and, in turn, indirectly impacting children through their parents. These were intertwined, as parents noted that living in a single-parent household had a financial and emotional impact. In this way, participant 11 stated [Original Spanish], “I believe that being a single mother is difficult because the responsibility falls on one person, both financially and emotionally, to raise the children.” Thus, subtheme 6, “lack of resources,” represents the scarcity factor in relation to a parent’s sense of security for themself and their children; having the means is not just about financial stability but also about protecting their children with the basic needs of food and shelter.

6. Subtheme 6: Lack of resources (financial barriers)

Lack of resources (financial burdens) is a barrier that prevents an individual from having financial stability due to a lack of resources. Lack of resources was classified as an interlevel subtheme, given that the challenges of single parenthood recur at the interpersonal level and closely mirror the issues identified in single-parent homes. Parents reported that financial barriers associated with childcare negatively impacted their resilience levels. Participant 6 stated, “To be honest, what really has hindered me is obviously a lack of resources. I should say so. Obviously, I was a single mother for a good four years before my partner and I got together, so support resources like daycare opportunities…” Therefore, parents describe childcare as a financial factor that directly impacts their resilience, as it is associated with the everyday care of their children. Participant 120 stated, “…If you don’t have the ways and the means of caring for the child… Well, maybe, like, you know, like finding a job or something, or you know, and stuff like that. So that would be like, you know, the stressful part about it.”

#### 3.2.2. SEM Interpersonal Level

- Theme 3: The Power of Close Relationships

This theme highlights the importance of supportive, close-knit relationships in a parent’s life. This theme indicates that social support functions as a critical buffer for parents, helping them manage stress and maintain more positive interactions with their children. Through meaningful discussions with parents at the interpersonal level, parents recharge and show up more effectively for their children through the support of parent friends and family. Only two subthemes arose for parents at the interpersonal level. Parents described external factors such as friends and family along with being part of PSGs (i.e., interlevel subtheme) as resilience builders through belonging and shared understanding.

7. Subtheme 7: Friends and family

Friends and family played a crucial role in supporting parents, providing both practical and emotional assistance. Friends and family helped parents find time for themselves and, in doing so, provided support that contributed to their resilience. Parents described their friends and family as providing aid to take a recharging break. For example, participant 5 mentioned, “Family members have been able to help me cope when I need to take a break or a breather.” Further, participant 4 mentioned how friends and family helped parents feel supported, stating, “What is helpful, I would say, is family support. Having that also supports.” This subtheme alludes to the saying, “It takes a village to raise a child,” and calls for understanding that the parenting mission is a heavy load not meant to be carried alone, and the vital role that friends and family have in sharing the weight of raising a child, offering support, guidance, and care along the way.

8. Subtheme 8: PSG interpersonal (inter-level subtheme)

At the interpersonal level, PSGs (i.e., interlevel subtheme) represent external protective (EP) factors that impact the individual through the collective parent group, making them an interpersonal EP factor. PSGs are described by parents as safe interpersonal yet external spaces, where they can share parental challenges, gain understanding, and receive mutual validation. Parents note that joining the PSG was not only beneficial for building connections but also practical in helping them cope with the everyday stress of parenting. Parent 13 mentioned, “For example, having a connection with other parents facing similar challenges, like sharing experiences and receiving emotional support. Practical support is basically like a peer-to-peer [parent] support group…” The PSG at the interpersonal level shows the importance of social connectedness and mutual understanding, that a sense of belonging eases the load of parenting. For example, participant 7 stated,

“… the most beneficial part of that to me was having the other parents to talk to, like, even outside of, like, the curriculum itself, just being able to be like, ‘Oh, this happened today,’ or like, oh, and she, you know, someone else is like, ‘Oh, my gosh, yes.’ Just that conversational support is huge for me.”

Further, another participant emphasized the role of PSGs from the perspective of social support for parents. In other words, PSGs represent an EP factor at the interpersonal level through the aspects of belonging and mutual support through parental connection.

#### 3.2.3. SEM Community Level

- Theme 4: Community Resources as a Buffer

This theme highlights the systemic, community-level impact of PSGs on parent resilience (i.e., interlevel subtheme), particularly within a child’s primary environments—school and home. PSGs were mentioned by parents as a means of emotional support, but this time at the community level, associated with schools. Parents described how PSGs helped them understand that other parents are going through the same situations (reducing isolation by normalizing shared experiences), which, in turn, strengthens parental coping and engagement. While PSGs were previously described as sources of emotional support, parents’ accounts at this level suggest a broader function: PSGs act as connectors between families and their surrounding community systems. This suggests that PSGs could act as protective organisms, helping build parental resilience at the community level. Participant 8 emphasized, “Like the sessions that I went to, like finding a group that you could go to where you could find similar parents in your situation, and you have that support.”

9. Subtheme 9: PSG community (interlevel subtheme)

To better explain, the PSG at the community level was described by parents as an “eye-opening experience” into understanding the impact of having awareness on community resources and how PSGs could fill the community gap of better school experiences (subtheme 10), improved school communication (subtheme 11), and protection (subtheme 12). Participant 11 noted [Original Spanish], “Well, the [parent] group really opened my eyes. It helped me a lot. Not all schools provide that kind of support to parents.” This subtheme highlights the negative impact of ineffective communication about community resources, which—although limited—could still help ease the burden on parents facing adversity. For example, participant 15 recalled various aspects of the PSG, noting how, at the community level, it provided not only emotional support but also guidance in accessing resources, stating:

[Original Spanish]: “But the program really seems to me like a spectacular program and, above all, for me, it was a great support, because I had just arrived. I didn’t know anyone. I didn’t speak the language. I didn’t know how to get around in the country, and the girl who helped me didn’t just provide emotional support with my child, but also created that connection with the community...”

- Theme 5: Collaboration That Works: Parents navigating the school system

An important theme at the community level concerned the disconnect between parents’ perspectives and the school system. This theme captures the pain and frustration that both parents and children may experience at the hands of the school system, a system developed to educate the leaders of tomorrow. Further, this theme points to a broader structural misalignment in how schools and families understand their respective roles in supporting children’s education. The school system—intended as a key developmental support for children—can become a source of emotional strain when communication and collaboration between the school and families are limited or ineffective. Overall, Theme 5 indicates a need to move beyond the community level and build more systemically aligned collaboration that actively bridges the gap between families and schools.

10. Subtheme 10: Adverse experiences at school

Adverse experiences at school refer to distressing events that occur to a child or parent within the school environment. This highlights the systemic issues in schools (i.e., SEM community level) where parents experience incidents of labeling, misdiagnosis, and even physical harm, but how, through parental reflection, accountability, and support, children can thrive despite the school system. Participant 24 explained the negative experiences at the hands of the school system, which negatively impacted both the parent and child:

“…my son has attended seven different elementary schools, and at each one, he experienced some form of trauma. Every time I met with school staff; the complaints were the same: ‘These kids are so bad. They’re disrespectful. They’re angry.’ It’s a pattern—one I’ve seen repeated at all seven schools in our community. It’s like every child is being labeled…What really helped was taking responsibility for my own actions—apologizing to him for the things he’s seen, for the ways I may have failed him—and working to help him heal… I believe more parents need to do that: take accountability and help their children heal from what they’ve experienced.” This theme sheds light on the realities of many parents who struggle due to distressing events at school, and the truly negative impact of those experiences to parent–child emotional well-being.

11. Subtheme 11: School is not accessible

Parents’ lack of access to the school highlights the communication barriers that impact their ability to receive timely and meaningful information about essential updates about their children, such as academic progress and well-being. Many parents mentioned the difficulty of maintaining communication with the school, as the school is not inclined to keep parents informed. Participant 22 described their sense of school connection as a negative feeling, stating, “What I feel here, in this part of [U.S State removed], in this area where we are, is that if you don’t ask for a meeting with the teacher, an entire year can go by and you won’t find out anything.” This subtheme is important, as it points to the need to improve the U.S. school systems through shared responsibility and mutual communication for the well-being of the child population.

12. Subtheme 12: School plays a role in protecting families

Parents emphasized the important role schools play in preventing and mitigating ACEs by supporting and protecting families. Schools would be an external, trusted factor in parent resilience through their ability to observe a child’s emotional and behavioral changes, helping to recognize signs of ACEs, and schools have a responsibility to intervene by reporting. Participant 22 recommended that schools at the community level be involved in protecting families, stating:

“[Original Spanish]: I think, in terms of policy, what I would like the most is for there to be institutions that really... either within the schools or at the community level, places that could help parents cope with these situations in the best way possible, because these are challenges for education.”

Schools can be a frontline defense against preventing and mitigating ACEs. Schools could play a crucial role in improving a parent’s resilience, but also play a crucial role in leading the communities to prevent and mitigate ACEs through communication and education. This subtheme highlights gaps in school-to-parent communication and in ACE community awareness, which are key to increasing a child’s developmental odds.

#### 3.2.4. SEM Societal Level

- Theme 6: Parenting Through a Cultural Lens

This theme explores how culture shapes families’ perceptions of parenting resilience along with effective child development, drawing on their own cultural upbringing. Rather than reflecting only on their childhood, parents reflected on what ideas or norms they considered effective from their culture. Thus, parents reflected on the norms and beliefs they uphold and that they believe would benefit society in our current time. Hence, the subthemes at the SEM culture level include community solidarity and empathy-related emotions. The subthemes reflect the positive experiences that created a stable resilience-building environment for both the parent and child. This theme highlights how parental resilience can be strengthened by identifying the cultural strategies that work. In other words, rather than treating cultural strategies as embedded networks (where emotional norms operate as integrated systems of support), we can treat them as emerging from the intentional preservation and adaptation of cultural values that promote empathy and shared responsibility for child development.

13. Subtheme 13: Community solidarity

Community solidarity refers to the sense of reciprocal support among community members, which helps build parental resilience by reflecting an external factor that enables them to overcome everyday obstacles. Some parents emphasized that in U.S. culture, parents need to have a sense of mutual support and responsibility within a neighborhood or community to help look out for one another’s well-being and to protect children through collaborative efforts by all community members. This subtheme can be linked to the PSG subthemes at the community level, and the positive impact that parents emphasized as providing them with support and a sense of belonging. This subtheme involves a positive change in child protection in U.S. society today by adopting a cultural approach of community solidarity. Participant 15 described community solidarity as a valuable way to help protect their children:

“[Original Spanish] You grow up in the community—that is, with the support and backing of the community, which I think is something incredibly valuable. It’s not just your own perspective, but also the perspective of your neighbor, who is also a friend and can help you, too. For example: “Hey, I saw that the child went somewhere they weren’t supposed to go’… You feel like everyone is offering support in the process of caring for and raising the child, and I think that can be really valuable in this country.”

14. Subtheme 14: Emotion x empathy

“Emotion x empathy” is associated with family support because it builds confidence to tackle everyday obstacles. Some parents noted that in their culture, empathy is driven by emotion and builds human connection, hence, “emotion x empathy.” Participant 15 stated that certain communities build strong family bonds, which, in turn, build support:

[Original Spanish] “Well, I think we Latinos are very empathetic. Also, we have a strong bond with our families, and that creates strong individuals. I mean, when you feel supported and accompanied by your family, you go out into the world knowing that no matter what happens, you have that strong connection—and I think that’s something we need to preserve.”

This subtheme emphasizes how positive emotion drives societal change and suggests that adopting the positive, empathetic norms of other cultures could help turn the U.S. into an empathetic population capable of protecting children and families. In other words, emotion is needed to drive positive change, and parents’ perspectives are shaped by their desire to improve their parenting for their children’s well-being.

- Theme 7: Change Through a Policy Lens: “Anything that protects them”

This theme captures how parents implicitly challenge institutional definitions of policy by centering safety, responsiveness, and accessibility as its core functions. This reframing suggests a disconnect between top-down policy design and the lived realities of caregiving. Further, this theme captures how parents envision “policy” focused on preventing and mitigating ACEs under subtheme 15: ACE prevention education. Parents understand policy not as a bureaucratic law or process, but rather simply “protection” under subtheme 16: “Child protection policies.” While the parents in this study are not lawmakers, they noted a clear call for policies to reflect their lived realities of caregiving and prioritize children’s well-being. These findings suggest that bridging the gap between institutional frameworks and parental expectations may be critical for designing policies that are both practical and impactful in addressing ACEs through education.

15. Subtheme 15: ACE prevention education

ACE prevention education is an initiative designed to teach children, families, and educators how to prevent and mitigate ACEs. This subtheme reflects the parents’ call for education for themselves, the school system, and the community. Parents and children make up the school system; their voices should be heard, and the federal level needs to change to provide proper child development through ACE prevention. Parents need ACE prevention education to be a shared responsibility of both parents and educators to identify ACEs and respond (implement protocols) that aid children experiencing adversity. Participant 17 stated:

“I think it’s very necessary to train teachers on identifying possible cases of abuse, violence, or mistreatment. I don’t know if those trainings exist, because I know they do in my country. I mean, teachers are constantly being trained on okay, signs of sexual abuse, and what the teacher can do. There are defined procedures, a protocol for reporting, where the teacher should report it, and where the school’s psychologist or social worker should report it…”

Parents not only desperately call for schools’ involvement in helping them protect children from adverse experiences, but also for ACE prevention education. This subtheme focuses on the parental need for ACE prevention as a tool to protect children. Parents call for equipping parents and the school system with ACE awareness, prevention, and mitigation, to help make a positive change in today’s society.

16. Subtheme 16: Child protection policies

Child protection policies are laws that focus on a child’s dignity and protection, including their interactions with friends and family. It is important to note that most parents emphasized their lack of policy and law knowledge, yet they knew that true child and family protection is enforced by the law. Parent 20 mentioned, “I think, in terms of policy, what I would like the most is for there to be institutions that really... either within the schools or at the community level, places that could help parents cope with these situations in the best way possible, because these are challenges for education.” This subtheme shows that parents’ experiences are exposed to systemic shortcomings that place children at risk. In other words, this subtheme suggests that despite parents’ efforts to care for and protect their children, they may lack adequate institutional support and resources.

## 4. Discussion

### 4.1. Overview/Summary of Key Findings

Given the key role resilience plays as the mediator or buffer to trauma exposures, it is of great importance to consider interventions that help strengthen parental resilience. This study aims to explore parental perspectives on how to promote resilience among parent–child dyads who seek ACE prevention and mitigation education across the levels of the SEM framework. The study’s findings hold promise for providing a foundation for developing a parent-resilience social ecological model (SEM) of ACE prevention and mitigation by exploring parents’ diverse perspectives on overcoming or adapting to adversity. Seven main themes across SEM levels, along with 16 subthemes, were identified, highlighting the pertinent factors of each level that impact parental resilience.

The individual SEM level captured a more proximal description of parental resilience fortification, as well as the factors that weaken parental intrapersonal resilience. Similar and consistent with parent self-reflection-focused subthemes at the individual level, Huynh, Kerr, Kim, Fourianalistyawati, Chang and Duncan [28] found that mindful parenting (MP) (or self-reflection) and parental reflective functioning (PRF) correlate with improvement in parent and child well-being; parenting behaviors, including greater emotional regulation; and reduced parenting stress. At the interpersonal level, close parent relations represent the sense of support parents require to balance out their everyday adversity. Consistent with our interpersonal findings, the mediation study by Dong, Yang, To, Yan and Shen [29] found that higher perceived social support from family, friends, and significant others was associated with positive parent–child relationships, including increased sense of meaning and ability for parents. At the community level, receiving support from both the community and schools provides a sense of safety and improves parent–child relationships. In line with our results on the impact of the school environment on parents’ resilience and, thus, child well-being, Butler, Quigg, Bates, Jones, Ashworth, Gowland and Jones [30] found that higher levels of support from family and school adults significantly improved child mental well-being, whereas lower levels were associated with a higher odd of low mental well-being. This indicates that as the home and school context improve, so does mental health well-being. Then, at the societal level, factors of culture and policy describe the positive aspects of the respondents’ culture and provide recommendations for policy change. Consistent with prior evidence noted by McBain, Levin, Matthews, Qureshi, Long, Schickedanz, Gilgoff, Kotz, Slavich and Eberhart [31] highlighting ACE education as a promising intervention within healthcare systems, the CDC [32] “Resource to Action” recommendation calls for preventing ACEs through collaboration across education and government services [33]. Building on existing literature, the present study adds an additional layer to current recommendations by demonstrating parental demand for ACE prevention education, which may indicate strong community-level acceptability and support broader implementation beyond clinical settings.

### 4.2. Interpretation of Findings in Relation to Existing Literature

Parent support groups (PSGs) are emphasized as parent resilience builders across the individual, interpersonal, and community SEM levels under Theme 1: Many Faces of Parent Resilience, Theme 3: The Power of Close Relationships, and Theme 4: Community Resources as a Buffer. Parents felt a sense of belonging and understanding when they realized they were not alone. It is important to emphasize that research on the impact of support groups on ACEs is severely limited; there is a clear gap in the literature regarding PSGs as an intervention to enhance parental resilience. However, we found that the pilot study showed that their pilot trauma-informed parenting group showed improvements in parents’ anxiety, stress, and emotional regulation. Although the existing literature on PSGs for parents, with a focus on parental resilience and ACE prevention and mitigation, is limited, studies from related populations were included because they provide the closest available evidence on PSG mechanisms. The following studies demonstrate improvements in psychosocial outcomes for PSGs in parent emotional regulation, stress reduction, and mental health well-being, which closely align with the processes and mechanisms identified in the present study.

The study by Grennan et al. (2022) [34] found that parents emphasized “I am not alone” as a result of their participation in a PSG. Thus, PSGs have a positive cross-level impact. At the individual level, the PSG helped parents regulate their own emotions (ground their emotions) through the reduction in isolation and learning to manage stress, while at the interpersonal level, parents emphasized the feeling of peer connection with other parents, and finally, at the community level, parents sensed community, access, and institutional support. Similarly, Lee and Choi (2023) [35] found that PSGs have the potential to make changes at the interpersonal, intra-individual, and sociopolitical levels. The socio-political term then intersects with social factors (e.g., culture) and politics (e.g., policymaking), helping parents understand systemic issues and advocate for their children and communities. Incorporating the social and political domains can help parents become critical thinkers, thereby strengthening their family systems through preventing and mitigating ACEs.

Further, the feasibility study by Nicula et al. (2023) [36] of a virtual parent-led support group of children with eating disorders (EDs) qualitatively reported that parents experienced less isolation, and quantitatively reported decreases in burden, unmet needs, and increased confidence (Nicula et al., 2023) [36]. Similarly, the study by Grennan et al. (2022) [34] found that parents with lived experience of having a child who had recovered from an ED found a PSG intervention helpful due to being able to share similar experiences and gain access to education, resources, and support. Somewhat similarly, the feasibility study conducted by Lee and Choi (2023) [35] with parents of youth with intellectual and developmental disabilities indicated that PSGs are a valuable way to promote parental empowerment. Further, a support group study by Sharma et al. (2022) [37] focused on reducing anxiety and stress among parents of children with autism and attention deficit hyperactivity disorder (ADHD). After participating in the support group, parents showed a significant reduction in stress and anxiety, as indicated by pre-test and post-test scores (*p* < 0.05) (Sharma et al., 2022) [37]. Future research should be focused on strengthening PSG frameworks (Grennan et al., 2022) [34]. Parents also highlighted the role of schools in facilitating parent group involvement.

School collaboration and accessibility were discussed at both the community and policy levels, under Theme 5: Parents navigating the school system and Theme 7: Change Through a Policy Lens: “Anything that protects them.” In our study, parents perceived poor school collaboration and communication. Many parents emphasized their reliance on the school system, noting that children spend the majority of their time in the school environment. Parents expressed a strong desire for meaningful communication and support from school communities. Research by Cui & Wen (2025) [38] found that school–home communication (SHC) positively predicts children’s enjoyment, success, and improved academic performance. Further, the study by Hummel et al. (2022) [39] found that a parent’s perception of the quality of school communication is positively associated with parent trust, which in turn predicts behavioral disorders and lower emotional symptoms of behavioral disorders in children.

While parents emphasized the importance of laws that protect children, they often lacked clarity regarding specific policies, their application, and the appropriate channels for enforcement. These discussions revealed a broader need for accessible policy knowledge, as well as the discomfort some parents experience when navigating or speaking about legal frameworks. Tuck (2012) [40] describes how parents who are under-resourced are more likely to be negatively affected or to fail due to policy. Furthermore, Tuck (2012) [40] describes a “deficit model” that overlooks crucial aspects of parental success and focuses on the absence of weaknesses within a family system. Future policy should focus on evidence-based research that leverages the strengths of family structures to effectively address weaknesses in child protection policies.

### 4.3. Implications for Future Research

Based on the study findings, parents are often navigating their own childhood trauma while trying to protect and provide for their children; however, various factors (single-parent home, limited economic means, and adverse experiences at school) hinder their ability to thrive under adversity. Furthermore, based on the findings of this study, PSGs may provide an important resource for parents of school-age children, specifically by institutionalizing trauma-informed principles into everyday school practices through the use of PSGs. In other words, trauma-informed care should be integrated into community-based services—particularly schools—through evidence-based strategies such as PSGs (Liu et al., 2024) [41]. This study provides several actionable recommendations. Specifically, schools could institutionalize mental health and well-being PSGs and teachers to support the prevention and mitigation of ACEs, and embed resilience-focused strategies to integrate in their respective communities. In other words, strategies such as developing emotionally safe learning environments and establishing clear protocols for staff training on trauma recognition, along with responding to students and families experiencing ACEs, would help prevent and mitigate ACEs.

From a policy perspective, our study’s findings suggest that policymakers should integrate interventions that help parents build resilience to support children’s development. Specifically, policymakers should provide funding for PSGs to be institutionalized to help shed light on systemic school issues that may be impacting the children. Further, parent–child mental health well-being policies should be embedded in the nation’s mental health strategies (through funding, collaboration, and alignment with mental health and resilience family support frameworks). At the global level, similar patterns have been observed, suggesting that the mental health stability of parents and their children is a wider systemic issue, rather than a local context-specific phenomenon [42]. Therefore, policymakers advocate for parenting programs as a universal prevention strategy [43]. Further, this study highlights the importance of embedded support interventions within institutional frameworks for volunteers.

### 4.4. Strengths and Limitations

A strength of this study is its use of qualitative methods to assess unexplored parental perspectives on ACEs and resilience, guided by the socio-ecological model. Using the socio-ecological model to guide the qualitative analysis of the parent ACE perspective informed the development of an initial multilevel parent-resilience framework.

Incorporating the SEM levels associated with ACE prevention and mitigation provides a framework for developing culturally grounded tools that support families in critically reflecting on potentially harmful cultural beliefs (e.g., the normalization of severe physical punishment), while also promoting strength-based practices (e.g., respect) that align with both cultural values and child well-being. These tools should be developed to help families critically reflect on harmful beliefs and help protect child safety and well-being, both physically, mentally, and emotionally. A key limitation of this study is the sampling frame, which included only parents who voluntarily participated in the community-based mental health organization’s parent support group. Thus, participants were recruited from an existing PSG, potentially introducing sampling bias, as the group is likely to differ from the broader population in terms of parent help-seeking behavior and motivation. The findings may not be fully generalizable to individuals who are not engaged in similar support services. Furthermore, the reliance on self-report measures may have introduced bias, including recall inaccuracies and subjective interpretation of questions. Finally, an additional limitation of this study is that the coding and primary analysis were conducted by a single coder, which may have influenced data interpretation and theme development. To enhance the trustworthiness and rigor of the analysis, we engaged in regular peer debriefing and discussion among the research team. Although these parents possess a uniquely informative perspective, future studies should replicate this methodology and framework in at-risk populations who are not seeking counseling.

## 5. Conclusions

This study identified various factors that both support and impede parental resilience at the SEM level, based on the lived experiences of parents seeking ER- and ACE-related support. The results provide a parent-informed framework for parental resilience, based on parental perceptions of areas of their lived experiences and backgrounds (e.g., culture or upbringing) that they believe would improve society. Thus, the resilience dimensions at the individual, interpersonal, community, and societal levels (including policy and culture) form the initial framework for parent resilience. The findings from this study hold promise for informing the development of trauma-informed health promotion as part of a parent-resilience framework that incorporates ACE prevention and mitigation.

## Figures and Tables

**Table 1:** Demographic characteristics and ACE scores (n = 20) *.

Characteristics	N	%	Mean	SE	SD
Gender					
Female	17	85%			
Male	3	15%			
Education					
GED	1	5%			
High school diploma	8	40%			
Bachelor’s degree	8	40%			
Master’s degree	3	15%			
Hispanic					
Non-Hispanic	10	50%			
Hispanic	10	50%			
White					
Non-White	4	20%			
White	16	80%			
African American					
Non-African American	14	70%			
African American	6	30%			
ACE stratification					
Zero ACEs	3	15%			
Less than 4 ACEs	12	60%			
Greater than 4 ACEs	5	25%			
ACE Score			2.8	0.63	2.80

**Notes:** N = 21 completed parent interviews. N = 20 * completed demographic survey. ACE range 1 to 10 (4 > ACEs = high ACEs). Participants could select more than one racial and/or ethnic category; therefore, percentages may exceed 100%.

**Table 2:** Parents’ definitions of parent support group themes and subthemes, organized by socio-ecological model level.

**Individual Level**	
Theme 1: The Many Faces of Parental Resilience: Represents the parents’ internal fortress of inner strength and emotional resilience shaped by their evolving experiences.	
Subtheme 1. Faith	Parents noted how their everyday spiritual practices, such as prayer, help them remain positive and stronger during difficult times.
Subtheme 2. Reflecting on their own childhood	Parents used a reflective approach to parenting focused on examining their own childhood experiences, including the happiness or pain they experienced, in an effort to create a better experience for their children.
Subtheme 3. For their children	Parents mentioned how their resilience stems from the love they have for their children, as they wish to model healthier, more efficient ways to cope.
Subtheme 4. Emotional regulation	Parents emphasized not only recognizing and understanding their emotions but also controlling their response in a healthy way to create a supportive household environment for the child.
Subtheme 5. Parent support group (PSG) individual ***	Parents reflected on how the PSG helped them feel as though they are not alone in parenting challenges, with positive influence from the individual level (e.g., coping and self-efficacy).
Theme 2: Resilience forged in scarcity: Parents highlighted external structural challenges—such as single parenthood and financial hardship.	
Subtheme 6. Lack of resources (financial barriers)	Parents mentioned the emotional toll and stress from financial instability, single parenthood and lack of resources to help parents with childcare, economic stability, and overall maintenance of basic necessities.
**Interpersonal Level**	
Theme 3: The Power of Close Relationships: Parents emphasized their resilience development with the parent support groups (PSGs) and the power of close relationships.	
Subtheme 7. Friends and family	Parents noted the emotional support and reassurance they received from “friends and family,” which helped them cope with everyday stress and increase or strengthen their resilience as parents.
Subtheme 8. PSG interpersonal ***	Parents reflected on the positive impact of the PSG in feeling connected with other parents on an interpersonal level (e.g., peer support).
**Community Level**	
Theme 4: Community Resources as a Buffer: Community resources helped parents maintain resilience, especially parent groups, which helped them feel understood and emotionally supported.	
Subtheme 9. PSG community ***	Having institutional support at the school and access to PSGs at the community level (e.g., social connectedness).
Theme 5: Parents navigating the school system: Parents emphasized the disconnect between parents’ perspectives and the school system, which negatively impacted the experiences of children at the school.	
Subtheme 10. Adverse experiences at school	Parents highlighted the challenges faced within the school system, emphasizing the need for parents to act to protect their children from negative school experiences.
Subtheme 11. School is not accessible	Parents faced communication barriers to receiving timely information about essential updates about their children, such as academic progress and well-being.
Subtheme 12. School playing a role to protect families	Parents pointed to the school’s ability to observe a child’s emotional and behavioral changes, helping to recognize signs of ACEs, and noting that schools can be a frontline defense to prevent and mitigate ACEs.
**Societal Level**	
Theme 6: Parenting Through a Cultural Lens: This theme reflects the influence of culture on how families understand parenting and effective child development based on how they were brought up.	
Subtheme 13. Community solidarity	Parents mentioned the need in U.S. culture to have a sense of mutual support and responsibility within a neighborhood or community to help look out for one another’s well-being, with all community members protecting children collaboratively.
Subtheme 14. Emotion x empathy	Parents noted that empathy is driven by emotion and builds human connection.
Theme 7: Change Through a Policy Lens: “Anything that protects them”: Parents envision policy focused on preventing and mitigating ACEs.	
Subtheme 15. ACE prevention education	Parents described the need for ACE prevention education as a shared responsibility of both parents and educators to both help recognize how to identify ACEs and respond (implement protocols) to children experiencing ACEs.
Subtheme 16. Child protection policies	Parents pointed out the importance of legal and institutional measures to help prevent ACEs and strengthen prosecution, enforcing strict penalties for all involved.

Notes: *** inter-level subtheme.

## Data Availability

The data presented in this study are available on reasonable request from the corresponding author. Access is restricted to ensure confidentiality and to protect the anonymity and privacy of study participants.

## Abbreviations

The following abbreviations are used in this manuscript:

SEM	Socio-Ecological Model
PSG	Parent Support Group
ACE	Adverse Childhood Experience
ER	Emotional Regulation

## Appendix A. Interview Guide: Building A Resilient Parent Socio-Ecological Model

**INTERVIEW GUIDE**
**BUILDING A RESILIENT PARENT SOCIO-ECOLOGICAL MODEL**
***The questions are framed by the socio-ecological model (interview guide).***

**Informed consent** must be provided to the participant. Proceed to interview **[if participant verbally states to agree to participate].**

**Disclaimer:** If you feel uncomfortable at any time or a question makes you uncomfortable, we can stop the interview or skip the question at any time. The interview is under your control.

**Start Interview:** Now we are reading to begin [start with question 1].

**INDIVIDUAL**
***This level includes beliefs, attitudes, and behaviors that prevent violence/trauma.***

QUESTION 1. We are interested to learning what has helped you thrive in the face of adversity and what has not been helpful. [Resilience can be described as the ability to cope with stress (i.e., adaptability, coping, and ability to deal with everyday obstacles in a parent’s life.)] What has helped you build resilience as a parent? What is difficult for parents?

**INTERPERSONAL (MICROSYSTEM):**
***This level includes close relationships (peers, partners, or family members), along with parenting (parent–child communication) and family mentoring (promoting positive peer norms, promoting healthy relationships).***

QUESTION 2. What strategies or activities do you think would help a parent or caregiver improve their relationship with their child?
  - Probe: What kinds of activities benefit parent–child relationships? (These could be communication and quality time, work–life balance, etc.)
QUESTION 3. What strategies or activities do you think would help parents and caregivers strengthen the emotional support available to them? (Emotional support means feeling supported.)

**COMMUNITY (MESOSYSTEM):**
***Schools, workplaces, or neighborhoods can both increase risk and protect against violence and trauma.***

QUESTION 4. What kinds of community support do you think are most useful or impactful for parents? (Examples include faith-based or social events that are open for communication.)
QUESTION 5. In school settings, what strategies or activities could be promoted that would benefit parents?
QUESTION 6. In community settings, what community activities would improve a parent–child environment or relationship? (Examples include afterschool programs, parks and recreation programs, or free lunch programs.) Additional probe: Do you have any ideas or recommendations to engage parents who are less interested in community activities?

**POLICY AND CULTURE LEVEL:**
***This level includes social and cultural norms that may support or prevent violence and trauma and may include factors that contribute to equality as related to health, education, economic or social conditions.***

QUESTION 7. Which norms regarding a parent–child relationship from your culture do you think would benefit society and why?
QUESTION 8. What strategies or policies would you recommend to help society prevent child abuse and neglect (for example, would the strategies be economic-focused, safety-focused, or legally focused)?
